# Surgical Treatment of Tuberculous Pleural Empyema in Children and Adolescents: A Retrospective Cohort Study

**DOI:** 10.3390/pathogens15090926

**Published:** 2026-09-02

**Authors:** Dmitry B. Giller, Alexandr D. Chuishchev, Inga I. Enilenis, Vadim V. Koroev, Oleg S. Kesaev, Galina V. Shcherbakova, Patimat G. Gadzhieva, Ludmila P. Severova, Nadezhda I. Klevno, Alexey V. Kazakov, Olga P. Frolova, Olga V. Butylchenko, Alexandr N. Ilyukhin, Valeriya A. Basangova, Evelina Y. Morozova, Ivan I. Martel

**Affiliations:** 1 Department of Phthisiopulmonology and Thoracic Surgery Named After M.I. Perelman, Sechenov First Moscow State Medical University (Sechenov University), 119991 Moscow, Russiashcherbakova_g_v@staff.sechenov.ru (G.V.S.);; 2 Moscow Regional Clinical Anti-Tuberculosis Dispensary, 127055 Moscow Oblast, Russia; 3 National Medical Research Center of Phthisiopulmonology and Infectious Diseases, Ministry of Health of the Russian Federation, 127473 Moscow, Russia; 4 Department of Phthisiopulmonology, Pirogov Russian National Research Medical University, 117997 Moscow, Russia

**Keywords:** tuberculosis, pediatric surgery, pleural empyema, VATS, pleurectomy, lung decortication

## Abstract

Background: While non-specific pleural empyema in children can be successfully managed with conservative drainage and thoracentesis in 80–84% of cases, tuberculous pleural empyema (TB empyema) exhibits high resistance and frequently requires extensive thoracic surgery. The choice of surgical scope depends directly on disease stage and secondary complications. Methods: We conducted a retrospective cohort study of 85 pediatric patients (aged 4 to 17 years) with confirmed TB empyema treated between 1984 and 2022, and followed until 2025. Disease staging followed a predefined adaptation of the American Association for Thoracic Surgery (AATS) consensus classification. The study adhered strictly to STROBE guidelines, incorporating temporal stratification across three historical eras and standardized Clavien–Dindo complication reporting. This study describes a 38-year surgical experience at a tertiary pediatric phthisiosurgical center; non-operated patients successfully cured with exclusive chemotherapy were excluded. Results: Stage I empyema was identified in three patients, Stage II in seven, and Stage III in 75 patients (79 total procedures). Stage I–II patients underwent video-assisted thoracoscopic (VATS) debridement. In Stage III, advanced reconstructive interventions were required (39 VATS pleurectomies, 21 segmental resections, eight pleuropneumonectomies, six lobectomies, two thoracomyoplasties, two open resections, and one bronchus occlusion). Postoperative complications occurred in two patients (2.35% patient-level; categorized as Clavien–Dindo Grade IIIa and Grade IIIb), presenting as delayed lung re-expansion managed via secondary minor interventions. Overall 30-day postoperative mortality was 0.0%. Over a median follow-up of 48 months (database lock: 31 December 2025; 97.65% 36-month completeness), zero disease recurrences were documented (0.0%). Conclusion: In this tertiary surgical cohort, combined management incorporating stage-dependent surgical intervention achieved durable clinical cure. Early VATS debridement represents an effective option for Stage I–II patients who fail initial drainage, potentially reducing progression to a rigid fibrothorax and the subsequent need for extensive pulmonary resections.

## 1. Introduction

Tuberculous pleural empyema (TB empyema) is characterized by the accumulation of purulent exudate infected with Mycobacterium tuberculosis within the pleural cavity. Unlike non-specific parapneumonic empyema, the clinical course in pediatric patients is characterized by subtle subclinical symptoms, a high propensity for chronicity, and marked resistance to closed chest tube drainage alone.

In international literature, the American Association for Thoracic Surgery (AATS) consensus classification is widely utilized to categorize pediatric pleural infection [1]:

- Stage I (Exudative phase): Accumulation of low-viscosity exudate without significant pleural thickening or loculations.
- Stage II (Fibrinopurulent phase): Abundant fibrin deposition on visceral and parietal pleura, leading to loculation formation and early lung fixation.
- Stage III (Organizational phase): Formation of a rigid fibrous peel (fibrothorax) entrapping the lung, causing restrictive volume loss and persistent empyema cavities.

While non-specific Stage I–II empyema frequently resolves with conservative measures, tuberculous etiology induces accelerated fibrinogenesis due to high mycobacterial antigen concentrations. Chest computed tomography (CT) is vital for identifying characteristic features such as the “split pleura sign”, extrapleural fat proliferation, and pleural calcification.

Study Objectives and Specific Scientific Novelty:

To address key gaps in contemporary pediatric phthisiosurgery, this study provides a comprehensive evaluation of an expanded single-center cohort (N = 85) managed over a 38-year span (1984–2022). The primary objectives and novel contributions of this work include:

1. Temporal Era Analysis: Evaluating the 38-year evolution of surgical access (open thoracotomy vs. multi/uniportal VATS) and diagnostic modalities across three distinct historical eras.
2. Standardized Decision Matrix: Establishing a reproducible, stage-dependent surgical algorithm incorporating anatomical variables (destroyed lung, bronchopleural fistula [BPF], lobar cirrhosis, calcification, and drug resistance).
3. Functional and Structural Compensation: Quantifying long-term (36-month) respiratory function recovery (spirometry FVC/FEV1) and thoracic wall growth dynamics post-decortication and resection.
4. Pediatric “Salvage” Procedures: Reporting technical feasibility, indications, and perioperative safety for rare reconstructive interventions, including pediatric pleuropneumonectomies, VATS thoracomyoplasties, trans-sternal main bronchus occlusion, and double intercostal space pneumolysis.

## 2. Materials and Methods

### 2.1. Study Design and Patient Selection

This retrospective observational cohort study was approved by the Institutional Review Board (Ethics Committee) of Sechenov First Moscow State Medical University (Protocol No. 17–23 from 5 October 2023). Medical records of 85 children and adolescents aged 4 to 17 years treated for surgical TB empyema between 1984 and 2022 were thoroughly reviewed and followed until 2025. Given the sample size disparity between early (Stages I–II, *n* = 10) and late (Stage III, *n* = 75) disease, the study is structured as a descriptive observational cohort with exploratory secondary comparisons.

- Inclusion Criteria: Confirmed diagnosis of tuberculous pleural empyema (Stages I–III); age from 4 years to 17 years and 11 months; and complete surgical and medical history.
- Exclusion Criteria: Non-tuberculous pleural effusion; age 18 years or older; or exclusive conservative chemotherapy without surgical intervention.

Operational Criteria for TB Empyema Staging:

Preoperative staging was assigned based on contrast-enhanced chest CT by two senior thoracic surgeons and a radiologist blinded to long-term outcomes:

- Stage I (Exudative): Free-flowing non-loculated collection, pleural thickening <2 mm, absence of mediastinal shift. Intraoperative findings included serous fluid and a thin, transparent pleura. Histological findings revealed acute mesothelial hyperemia.
- Stage II (Fibrinopurulent): Loculated collection, moderate pleural thickening (2–5 mm), “split pleura sign”. Intraoperative findings demonstrated turbid exudate and loose fibrinous loculations. Histological findings showed PMN infiltration, with fibrinous exudate overlaying early epithelioid granulomas.
- Stage III (Organizational): Rigid fibrothorax, thick calcified/uncalcified peel (>5 mm), lung entrapment, rib crowding, extrapleural fat proliferation. Intraoperative findings revealed a dense fibrous sac and subpleural caseous debris. Histological findings demonstrated dense collagenous tissue with hyalinization, extensive caseating necrosis, and Langhans giant cells.

Diagnostic Verification Criteria:

Confirmed TB empyema required at least one of the following validated criteria: (1) isolation of Mycobacterium tuberculosis (MTB) on culture; (2) positive real-time PCR or GeneXpert MTB/RIF assay; (3) detection of acid-fast bacilli (AFB) on Ziehl-Neelsen microscopy; (4) histopathological verification of caseating granulomatous inflammation; (5) direct intraoperative visualization of gross caseopurulent exudate combined with microbiological confirmation in sputum/bronchial aspirate. This study adheres to the STROBE statement [2].

### 2.2. Cohort Evolution and Relationship to Previous Publications

This study expands upon an initial regional registry analysis of 82 patients treated up to 2017 published by Giller et al. in 2019 (Pirogov Russian Journal of Surgery) [3]. Key expansions include:

1. Cohort Expansion: Addition of 3 Stage III patients treated between 2018 and 2022 (increasing Stage III from 72 to 75 patients, and procedures from 76 to 79; total cohort N = 85).
2. Stage Disaggregation: Retrospective disaggregation of 10 early-stage cases into Stage I (*n* = 3) and Stage II (*n* = 7).
3. Diagnostic and Follow-Up Focus: Systematic evaluation of early VATS biopsy diagnostic yield (PCR/DST) and standardized 36-month follow-up across the expanded cohort.
4. Novel Materials: Entirely new clinical cases and figures (Section 4).

### 2.3. Surgical Decision-Making Protocol and Access Selection

Surgical strategy was guided by a standardized decision-making protocol:

- VATS Debridement and Partial Pleurectomy (Stages I–II): Indicated for non-organized exudative/fibrinopurulent collections with expandable underlying lung.
- VATS Pleurectomy with Decortication (Stage III): Indicated for organized fibrothorax with entrapped re-expandable lung, without underlying irreversible parenchymal destruction.
- Segmental Resection/Lobectomy with Decortication (Stage III): Indicated for empyema complicated by localized irreversible destruction (caseous cavities, lobar cirrhosis, persistent BPF).
- VATS Pleuropneumonectomy (Stage III—“Operation of Last Resort”): Indicated for total destroyed lung syndrome, subtotal cirrhosis with complete functional loss, or main bronchial stenosis.
- VATS Thoracomyoplasty/Muscle Flap Transposition (Stage III): Indicated for persistent rigid residual spaces following extensive resection.
- Trans-sternal Main Bronchus Occlusion: Indicated for persistent main bronchopleural fistula following failed prior ipsilateral surgery.
- Double Intercostal Space Thoracotomy: Indicated in severe recurrent fibrothorax with massive extrapleural calcification requiring dual exposure (5th and 7th/8th spaces).

Surgical Access Classification: VATS was the primary default approach. Single-access hybrid VATS minithoracotomies (6 cm incision utilizing video optics without separate trocars) were grouped under the composite “VATS/Hybrid VATS” operational category.

### 2.4. Temporal Stratification (Surgical Eras)

To account for technological evolution across the 38-year duration (1984–2022), the cohort was stratified into three eras:

1. Pre-VATS/Open Era (1984–1999, *n* = 32): Open thoracotomy, linear tomography, AFB microscopy, and solid cultures.
2. Transition Era (2000–2010, *n* = 31): Chest CT, early video thoracoscopy, qualitative PCR, and early mechanical staplers.
3. Modern VATS and Molecular Era (2011–2022, *n* = 22): Standardized multi/uniportal VATS, real-time PCR, rapid molecular DST (MDR/Pre-XDR), endostaplers, and modern pediatric ICU care.

### 2.5. Antituberculosis Chemotherapy Protocol

All the patients received chemotherapy managed under Directly Observed Therapy (DOT) inpatient protocols [4]:

- Drug-Susceptible TB: Standard 4-drug intensive phase (HRZE/S) for 2–3 months and followed by 2-drug continuation (HR) for 6–9 months (total: 9–12 months).
- Drug-Resistant TB (MDR/Pre-XDR): Individualized second-line regimens (Fluoroquinolones, Bedaquiline, Linezolid, Cycloserine, and Amikacin) for 18–24 months based on rapid PCR/culture DST.
- Postoperative Regimen Modification: Operative tissue PCR DST allowed regimen optimization within 48–72 h in 14 patients (16.5%).

### 2.6. Variable Definitions and Study Outcomes

- Primary Composite Endpoint (“Clinical Cure”): Defined as simultaneous fulfillment of (1) complete symptom resolution; (2) complete lung re-expansion/cavity obliteration on CT; (3) persistent microbiological clearance (culture/PCR negativity); (4) surgical resolution (cessation of drainage, fistula closure); and (5) uninterrupted chemotherapy completion without relapse over 36 months.
- Intraoperative Complications: Defined as major vascular/airway injury, massive hemorrhage (>15% blood volume), anesthetic catastrophes, or unplanned conversion/extension.
- Postoperative Morbidity: Stratified using the modified Clavien–Dindo classification system.
- Secondary Outcomes: Operative duration, blood loss, ICU admission, mechanical ventilation duration, chest tube duration, hospital stay, and spirometry metrics (FVC%, FEV1%).

### 2.7. Follow-Up Protocol and Database Lock

Outpatient monitoring occurred at 3, 6, 12, 24, and 36 months postoperatively. The final institutional database lock date was established as 31 December 2025. Late mortality and relapses were cross-checked against official regional TB registries.

### 2.8. Statistical Analysis

Statistical processing was performed using IBM SPSS Statistics version 22.0. Continuous variables are expressed as Mean ± SD or Median [IQR]. Categorical data are presented as absolute numbers, percentages, and 95% Confidence Intervals (95% CIs) calculated via the Wilson score method. Comparisons utilized Mann–Whitney U, Kruskal–Wallis, Chi-square, or Fisher’s exact tests (*p* < 0.05).

## 3. Results

### 3.1. Baseline Cohort Characteristics and Verification

The study included 85 patients: 47 boys (55.3%) and 38 girls (44.7%), with a mean age of 11 ± 4 years (range: 4–17 years). All 85 patients (100.0%) had definitive diagnostic verification (Table 1).

No statistically significant demographic differences were observed across stages (*p* = 0.290 for age, *p* = 0.279 for sex); however, these secondary comparisons were limited by small early-stage sample sizes (Table 2).

Baseline Comorbidities, Nutritional Status, and Complications:

Assessment of baseline systemic status revealed severe malnutrition (BMI-for-age Z-score < −2) in 32 patients (37.6%, 95% CI: 27.9–48.4%), occurring exclusively in Stage III cases (32/75, 42.7%). Moderate-to-severe normocytic anemia (hemoglobin < 100 g/L) was identified in 28 patients (32.9%). Preoperative nutritional support (high-protein oral supplementation or enteral nutrition) was administered for a median of 14 days (IQR: 10–21) prior to reconstructive surgery.

Bronchopleural fistulas (BPF) were identified in 11 Stage III patients (12.9%, 95% CI: 7.4–21.7%). Presence of a BPF directly governed the surgical choice: six patients underwent lobectomy or segmental resection, four underwent pleurectomy with direct bronchial suture and intercostal muscle flap reinforcement, and one patient with persistent main BPF required trans-sternal occlusion of the main bronchus. Concomitant extrapulmonary tuberculosis (peripheral lymphadenitis or skeletal TB) was present in 14 patients (16.5%). All 85 patients were HIV-negative (100.0%) and had no underlying primary immunodeficiencies.

### 3.2. Drug Resistance and Chemotherapy Outcomes

Drug susceptibility testing identified resistant strains in 25 patients (29.4%), including 19 cases of MDR/Pre-XDR TB (22.4%). Operative DST prompted immediate second-line regimen optimization in 14 patients (16.5%). Microbiological conversion of sputum and pleural exudate was achieved in 100.0% of patients at a median of 2.5 months postoperatively (Table 3).

### 3.3. Temporal Stratification Across Historical Eras

Stratification across study eras demonstrated a marked transition toward minimally invasive access (*p* < 0.001) and molecular diagnostics (*p* < 0.001), while complication rates (*p* = 0.721) and mortality (0.0%) remained stable (Table 4).

### 3.4. Preoperative Treatment Duration and Disease Progression

Longer preoperative conservative therapy was significantly associated with Stage III disease at surgery (median 5 months [IQR: 4–7] for Stage III vs. 1 month [IQR: 1–1] for Stage I and 1 month [IQR: 1–2] for Stage II, *p* < 0.001). Serial CT imaging in 12 un-operated patients objectively documented stage progression during protracted conservative management (three patients progressed from Stage I to II over 1–2 months; nine patients progressed directly from Stage I to Stage III over 4–10 months; mean 7 ± 2 months).

### 3.5. Structure of Surgical Procedures

In Stage III (*n* = 75), a total of 79 formal surgical interventions were performed (71 patients underwent one operation; four patients required two operations). Minimally invasive VATS approaches predominated (76/79 procedures, 96.2%) (Table 5).

### 3.6. Perioperative Metrics and Complications

Operative duration, blood loss, chest tube duration, and hospital stay were significantly lower in early VATS stages (*p* < 0.001). Spirometry in cooperative children (*n* = 68) demonstrated marked FVC improvement at 1 year (*p* < 0.001) (Table 6).

Postoperative complications occurred in two patients (2.35% patient-level; 2.53% procedure-level), categorized as Clavien–Dindo Grade IIIb (Case #1, requiring re-VATS under general anesthesia) and Grade IIIa (Case #2) (Table 7). Zero major intraoperative complications or 30-day deaths occurred (0.0%).

### 3.7. Long-Term Follow-Up and Recurrence

With the database locked on 31 December 2025, median follow-up was 48 months (IQR: 36–60; range: 14–144 months). Full 36-month follow-up was completed in 83 of 85 patients (97.65%, 95% CI: 91.8–99.4%); two patients were lost to follow-up at 14 and 20 months due to family relocation. Zero disease recurrences (0.0%, 95% CI: 0.0–4.3%) and zero late out-of-hospital deaths were recorded. All 85 patients (100.0%) fulfilled the composite criteria for clinical cure.

## 4. Case Reports and Illustrative Materials

### 4.1. Figure 1: Loculated Stage II Tuberculous Empyema (Patient K., 11 Years Old)

An 11-year-old boy presented with a loculated pleural effusion following one month of ineffective conservative treatment and therapeutic pleural punctures at a regional hospital. Pleurectomy with lung decortication was performed via a minithoracotomy approach. The imaging data for this clinical case are divided into two separate graphic files:

Panel A: Preoperative chest radiograph demonstrating a large left-sided loculation. Panels B, C, and D: Intraoperative views showing stages of pleurectomy and lung decortication performed via a minithoracotomy approach.

Panel E: Gross specimen of the loculation in cross-section, demonstrating caseopurulent contents. Panel F: Follow-up chest radiograph obtained on postoperative day 5, showing complete lung re-expansion and absence of pleural effusion.

**Figure 1:**
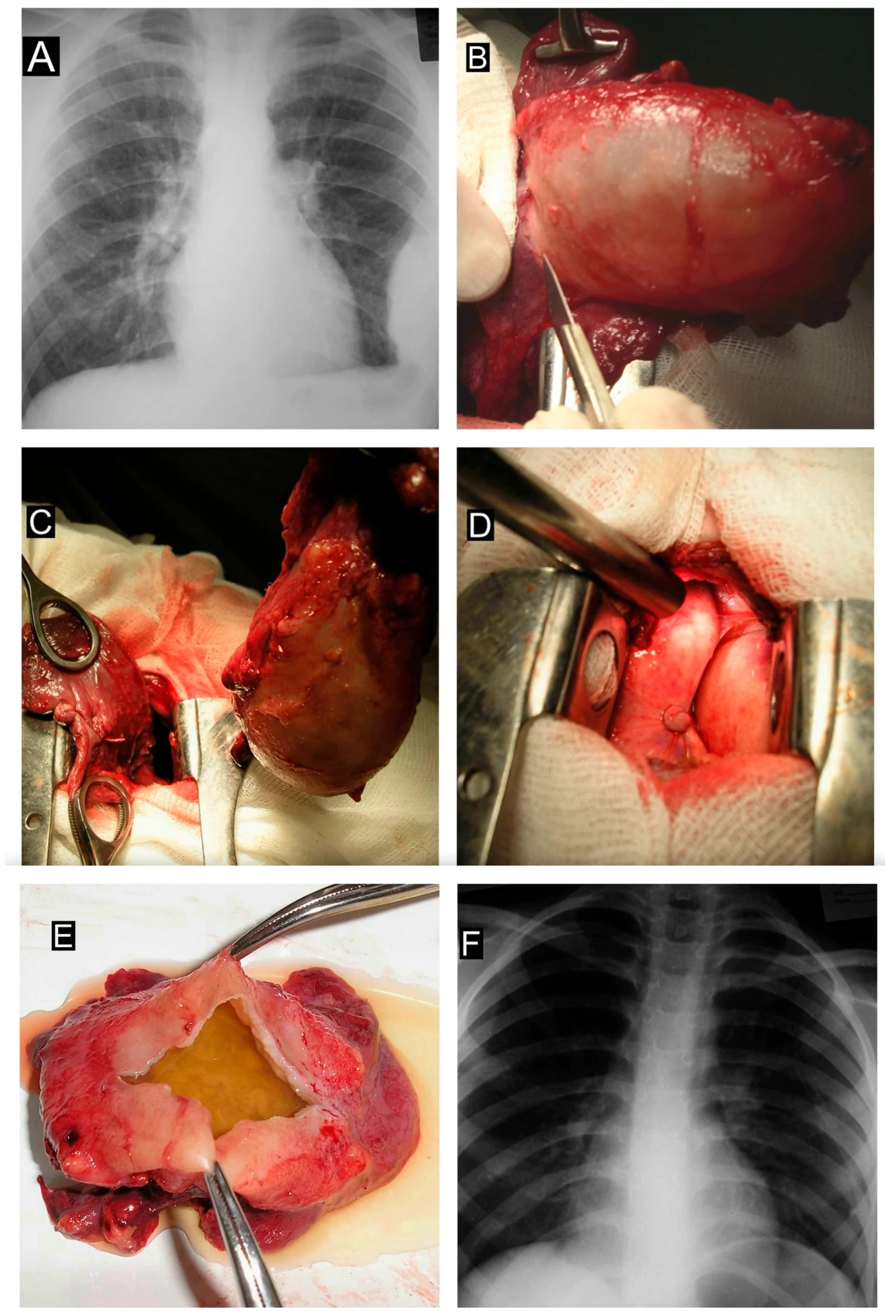
Case 1 (ID #18, Male, 11 yrs, Stage II, 2012): Illustrates the decision node for single-access hybrid VATS minithoracotomy (6 cm) for loculated fibrinopurulent empyema. Uncomplicated recovery; complete lung re-expansion achieved on day 5. Panel (**A**): Preoperative chest radiograph demonstrating a large left-sided loculation. Panels (**B**–**D**): Intraoperative views showing stages of pleurectomy and lung decortication performed via a minithoracotomy approach. Panel (**E**): Gross specimen of the loculation in cross-section, demonstrating caseopurulent contents. Panel (**F**): Follow-up chest radiograph obtained on postoperative day 5, showing complete lung re-expansion and absence of pleural effusion.

### 4.2. Figure 2: Total Chronic Stage III Tuberculous Empyema with Secondary Pulmonary Cirrhosis (Patient M., 4 Years Old)

A 4-year-old girl presented with intrathoracic tuberculous lymphadenitis complicated by total chronic left-sided TB empyema following 10 months of ineffective chest tube drainage. The imaging archive for this case is divided into two parts:

Panels A and B: Chest computed tomography (CT) scans demonstrate a total left-sided loculation and associated lung collapse. Panel C: Three-dimensional (3D) CT reconstruction of the lungs and tracheobronchial tree showing signs of left lung cirrhosis. Panel D: Intraoperative debridement.

Panel E: Pleurectomy with partial upper lobe resection. Panel F: 3D CT reconstruction at 1 month postoperatively, demonstrating incomplete lung re-expansion due to pulmonary fibrosis. Panel G: Chest radiograph at the 2-year follow-up, showing marked compensatory hypertrophy. Panel H: Clinical photograph of the child demonstrating the absence of chest wall deformity.

**Figure 2:**
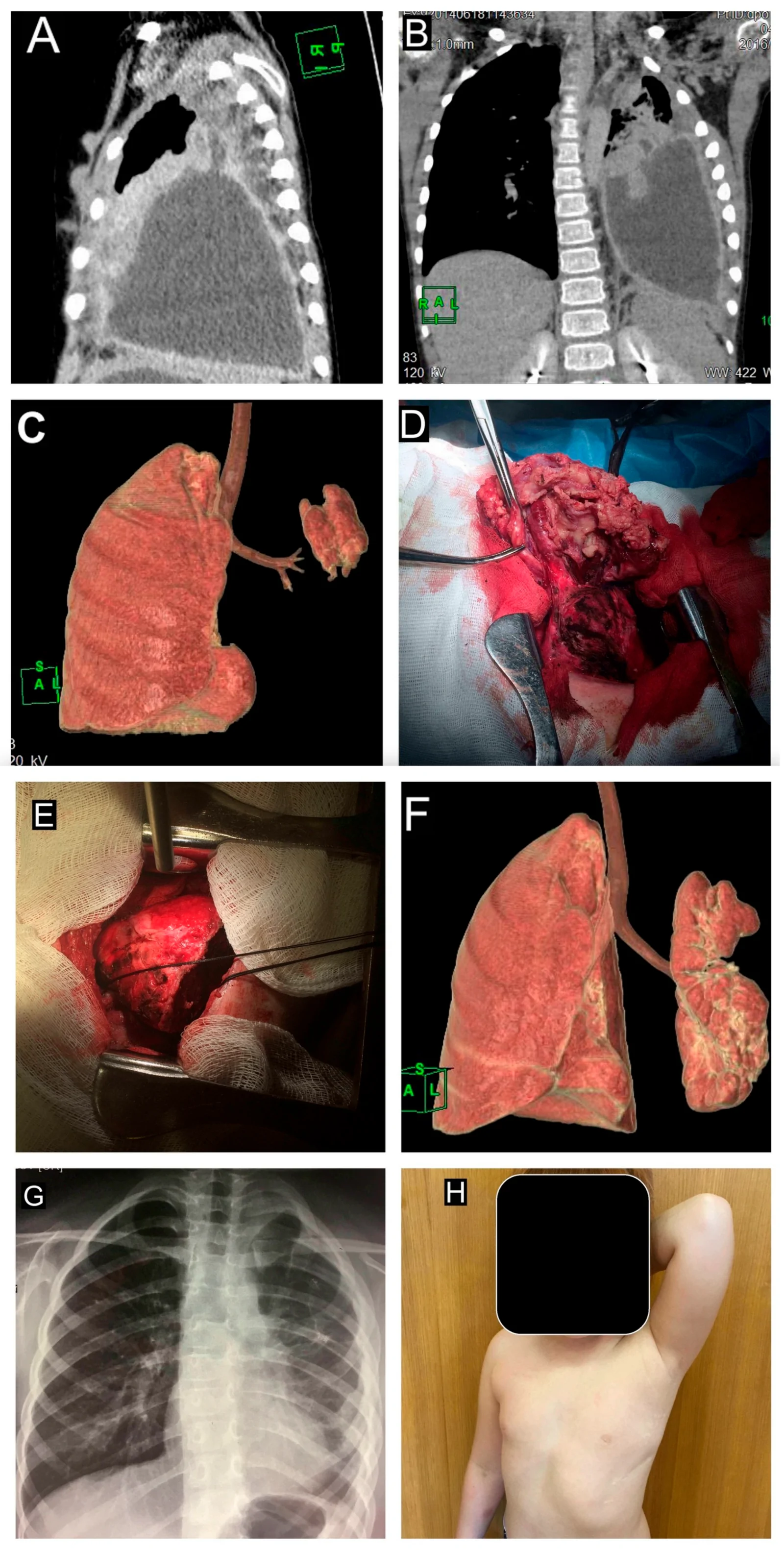
Case 2 (ID #79, Female, 4 yrs, Stage III, 2020): Illustrates the decision node for VATS lower lobectomy with pleurectomy in a young child with total empyema and secondary lobar pulmonary cirrhosis. Panels (**A**,**B**): Chest computed tomography (CT) scans demonstrate a total left-sided loculation and associated lung collapse. Panel (**C**): Three-dimensional (3D) CT reconstruction of the lungs and tracheobronchial tree showing signs of left lung cirrhosis. Panel (**D**): Intraoperative debridement. Panel (**E**): Pleurectomy with partial upper lobe resection. Panel (**F**): 3D CT reconstruction at 1 month postoperatively, demonstrating incomplete lung re-expansion due to pulmonary fibrosis. Panel (**G**): Chest radiograph at the 2-year follow-up, showing marked compensatory hypertrophy. Panel (**H**): Clinical photograph of the child demonstrating the absence of chest wall deformity.

### 4.3. Figure 3: Total Pneumolysis via a Double Intercostal Space Incision in Stage III Disease (Patient S., 17 Years Old)

A 17-year-old adolescent presented with recurrent organizing empyema following ineffective VATS performed at another institution. To achieve total pneumolysis, a surgical approach via a double intercostal space incision (the fifth and seventh intercostal spaces) was utilized. The illustrative material for this case is divided into two sections:

Panel A: Preoperative chest CT scan demonstrating massive multiloculated loculations. Panels B, C, and D: Intraoperative stages of dissecting the dense fibrous peel from the pulmonary surface.

Panel E: Intraoperative view of the surgical field via the double intercostal space approach. Panel F: Placement of three silicone chest tubes directed towards the apex, posterior sinus, and anterior sinus. Panel G: Gross specimen of the dense pleural sac, completely excised *en bloc*. Panel H: Chest radiograph obtained prior to hospital discharge.

**Figure 3:**
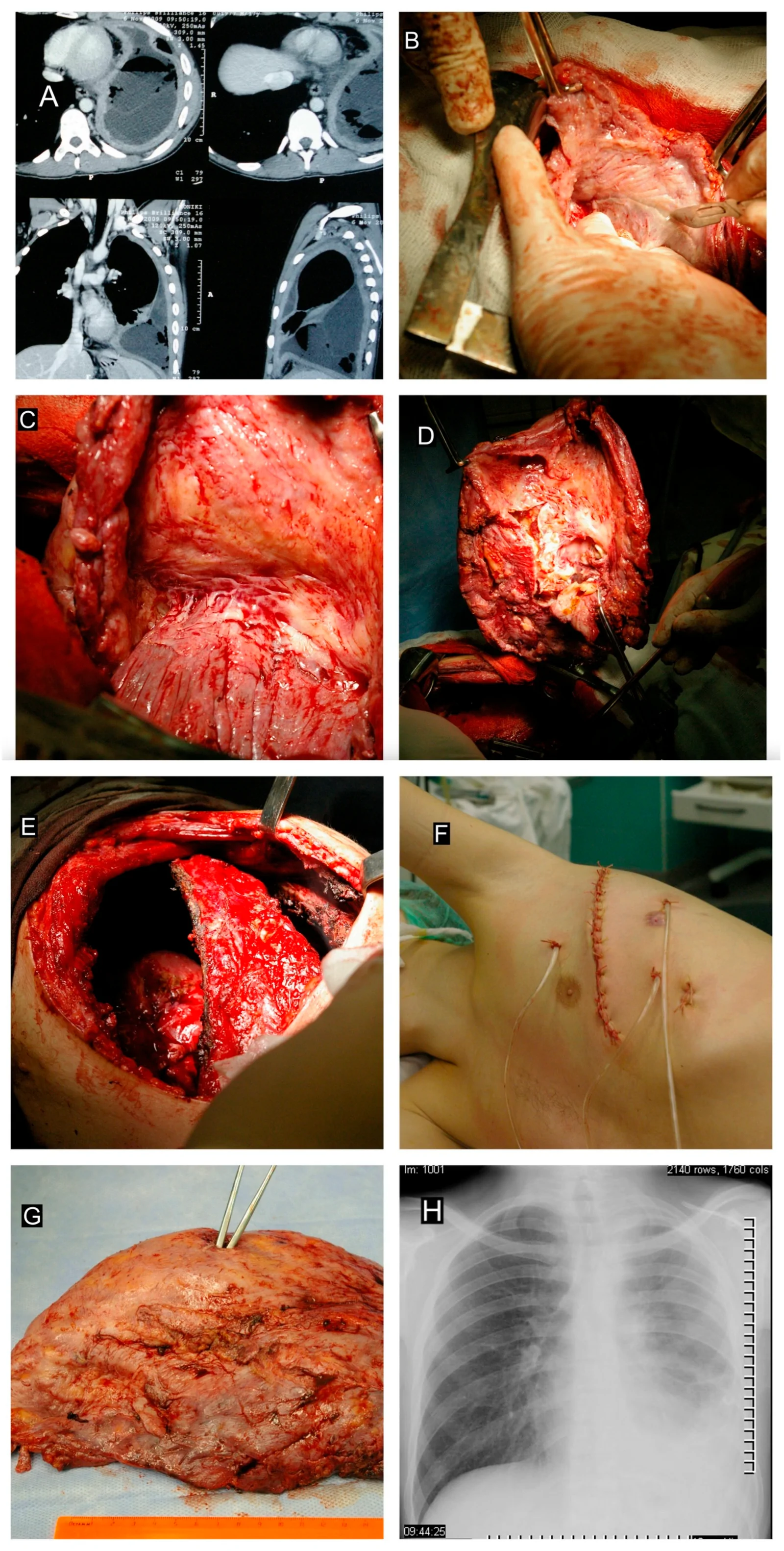
Case 3 (ID #83, Male, 17 yrs, Stage III, 2021): Illustrates the decision node for double intercostal space thoracotomy (5th and 7th spaces) for massive recurrent fibrothorax requiring complete extrapleural pneumolysis. Panel (**A**): Preoperative chest CT scan demonstrating massive multiloculated loculations. Panels (**B**–**D**): Intraoperative stages of dissecting the dense fibrous peel from the pulmonary surface. Panel (**E**): Intraoperative view of the surgical field via the double intercostal space approach. Panel (**F**): Placement of three silicone chest tubes directed towards the apex, posterior sinus, and anterior sinus. Panel (**G**): Gross specimen of the dense pleural sac, completely excised *en bloc*. Panel (**H**): Chest radiograph obtained prior to hospital discharge.

### 4.4. Figure 4: Tuberculous Empyema Complicated by Calcified Constrictive Pericarditis (Concretio Cordis)

This section presents a severe complication of advanced tuberculosis involving extension of the inflammatory process to the pericardium, with subsequent calcification (“armored heart”). All stages of the condition are illustrated in a single multipanel figure:

Panel A: Chest CT scan (arrows indicate the dense calcified shell encasing the myocardium and the loculated empyema along the right pleura). Panel B: Surgical excision of the rigid, calcified pericardial layer. Panel C: Intraoperative view of the surgical field following subtotal pericardiectomy, demonstrating the liberated pulsating myocardium. Panel D: Gross specimen of the excised tissue (top: empyema sac; bottom: stony calcified pericardium).

**Figure 4:**
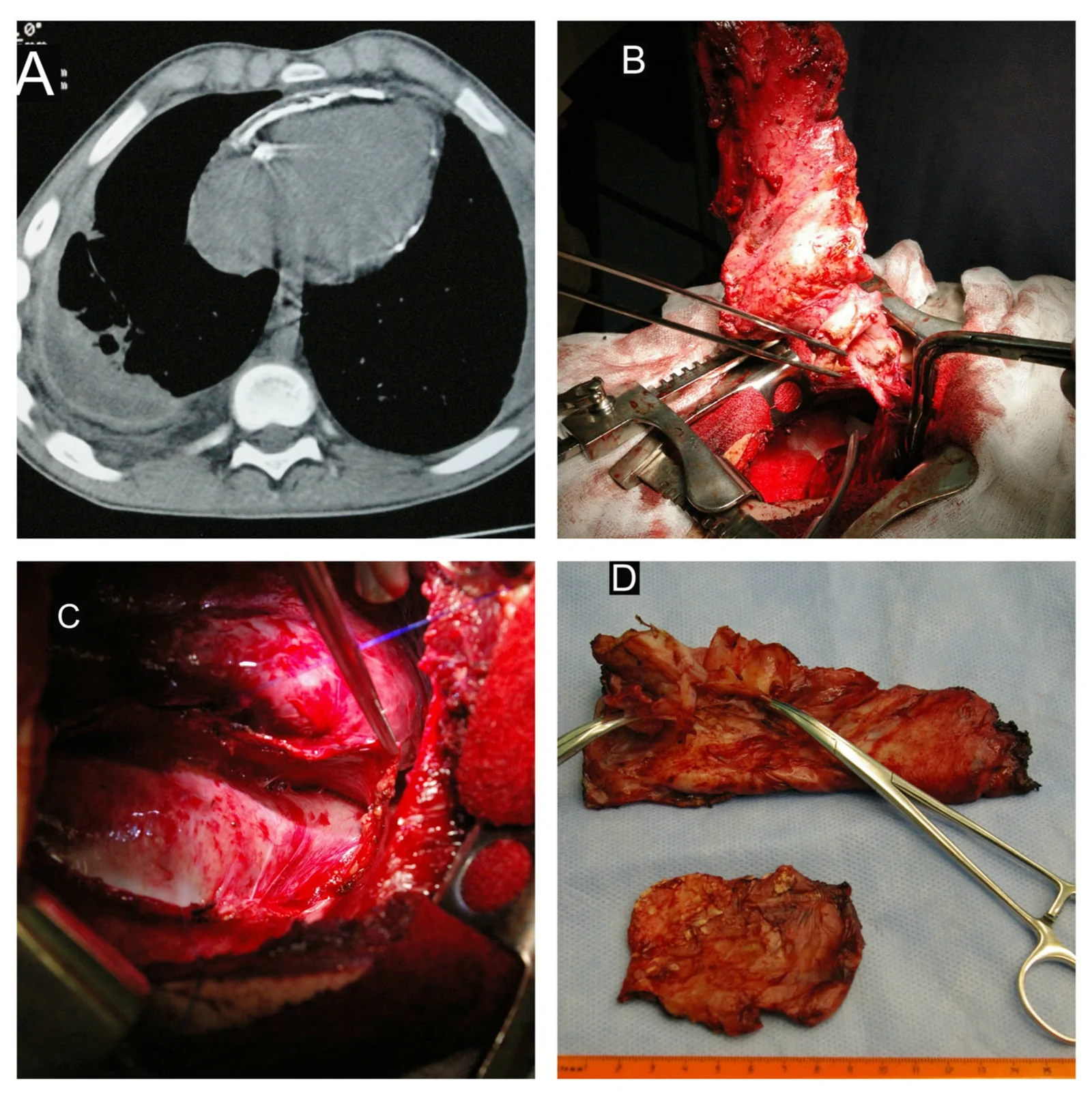
Case 4 (ID #64, Male, 15 yrs, Stage III, 2018): Illustrates a rare decision node—VATS decortication combined with subtotal pericardiectomy for Stage III empyema complicated by calcified constrictive pericarditis (“armored heart”). Relieved both lung entrapment and right heart constriction. Panel (**A**): Chest CT scan (arrows indicate the dense calcified shell encasing the myocardium and the loculated empyema along the right pleura). Panel (**B**): Surgical excision of the rigid, calcified pericardial layer. Panel (**C**): Intraoperative view of the surgical field following subtotal pericardiectomy, demonstrating the liberated pulsating myocardium. Panel (**D**): Gross specimen of the excised tissue (top: empyema sac; bottom: stony calcified pericardium).

## 5. Discussion

While international consensus guidelines for non-specific parapneumonic empyema prioritize conservative drainage and intrapleural fibrinolytics [5,6,7], our clinical observations in this surgical cohort suggest that exclusive reliance on conservative chest tube drainage often fails to halt disease progression when tuberculous etiology is present [8,9].

Pathogenetic differences determine distinct rates of progression. High concentrations of mycobacterial antigens within the pleural space induce procoagulant factor expression, accelerating fibrinogenesis [10]. As noted by Lee S.F. et al., conventional empyema strategies target acute purulent foci [5], whereas tuberculous empyema demands combined surgical debridement and targeted multidrug chemotherapy [11,12].

Pathophysiological Mechanisms of Tuberculous Pleural Organization:

Accelerated pleural organization in tuberculous empyema is mediated by complex interactions among mesothelial injury, a persistent mycobacterial antigen load, and an imbalance within the local fibrinolytic cascade. Elevated intrapleural concentrations of Mycobacterium tuberculosis antigens stimulate resident mesothelial cells and infiltrating macrophages to upregulate Tissue Factor (TF) expression while simultaneously inducing the massive production of Plasminogen Activator Inhibitor-1 (PAI-1). This marked suppression of intrapleural fibrinolysis promotes rapid fibrinous deposition. Subsequent sustained release of Transforming Growth Factor-beta 1 (TGF-β1) drives intense subpleural fibroblastic proliferation, extracellular matrix remodeling, and collagen deposition, ultimately transforming loose fibrinous loculations into a rigid, non-expansile fibrothorax.

In our series, early VATS debridement for Stage I–II disease provided major diagnostic and therapeutic advantages: rapid histological/PCR verification (confirming diagnosis in 50% of preoperatively unverified cases), immediate operative DST for MDR-TB management, and effective pleural decompression preventing rigid fibrothorax [13,14].

Despite these advantages, nearly 90% of children were referred in Stage III (organizing phase), requiring extensive decortications or resections. When lung entrapment was severe or anatomical planes were obliterated, specialized approaches—such as double intercostal space thoracotomy or salvage pleuropneumonectomies—were successfully utilized [15,16,17,18,19,20].

Following combined surgical intervention and targeted multidrug chemotherapy, all 85 patients in this cohort fulfilled the composite criteria for clinical cure, with zero 30-day mortality and no recurrences over 36 months of follow-up.

### Study Limitations

The primary limitation of this study is inherent selection and referral bias. By excluding patients who achieved complete resolution with conservative antituberculosis chemotherapy and simple drainage alone, this cohort cannot establish the true population-wide proportion of pediatric TB empyema requiring surgery. Furthermore, because non-operated control groups were not analyzed, these data cannot definitively prove that early VATS is superior to conservative medical management in all early-stage cases. Additional limitations include:

1. *Sample Size Imbalance:* Comparisons across stages were limited by small sample sizes in Stage I (*n* = 3) and Stage II (*n* = 7).
2. *Historical Record Bias:* Minor non-fatal intraoperative variances may have been under-reported in paper-based archives from the Pre-VATS era (1984–1999).

Our findings must be interpreted strictly as an observational evaluation of surgical decision-making and outcomes in patients referred to a specialized tertiary phthisiosurgical center due to non-resolving or advanced pleural disease.

## 6. Conclusions

1. In children and adolescents with persistent, loculated, or histologically/microbiologically confirmed tuberculous pleural empyema, early thoracic surgical evaluation should be considered, particularly when initial non-surgical management fails or organizing disease develops.
2. In this surgical cohort, perioperative morbidity, procedure extent, intraoperative trauma, and overall hospital duration were strongly associated with the disease stage at the time of surgical referral.
3. For patients referred with Stage I–II tuberculous empyema demonstrating persistent loculation or failed closed drainage, early video-assisted thoracoscopic surgery (VATS) debridement serves as a feasible, organ-preserving approach that may reduce progression to a rigid fibrothorax and help avoid secondary extensive pulmonary resections.

## Figures and Tables

**Table 1:** Diagnostic verification modalities and clinical phenotypes (N = 85).

Parameter/Feature	Absolute Number (*n*)	Percentage (%)	95% Confidence Interval (CI)
Verification Modalities			
— Histopathology (Caseating Granuloma)	81	95.3%	88.5–98.2%
— Real-Time PCR/GeneXpert MTB	30	35.3%	25.8–46.1%
— Culture Confirmation (MTB Positive)	27	31.8%	22.7–42.5%
— Microscopy (Acid-Fast Bacilli)	22	25.9%	17.5–36.4%
— Intraoperative Caseopurulent Visualization	85	100.0%	95.7–100.0%
Clinical Phenotypes			
— Acute Fibrinopurulent TB Empyema (Stages I–II)	10	11.8%	6.5–20.3%
— Chronic Organized TB Empyema (Stage III)	75	88.2%	79.7–93.5%
— Presence of Bronchopleural Fistula (BPF)	11	12.9%	7.4–21.7%

**Table 2:** Baseline demographic and clinical characteristics stratified by empyema stage (N = 85).

Characteristic/Variable	Stage I (*n* = 3) [95% CI]	Stage II (*n* = 7) [95% CI]	Stage III (*n* = 75) [95% CI]	Total Cohort (N = 85) [95% CI]	*p*-Value
Age (years, mean ± SD)	9 ± 5 [3.7–14.3]	13 ± 2 [11.1–14.9]	11 ± 4 [10.1–11.9]	11 ± 4 [10.1–11.9]	0.290
Sex (*n*, %)					0.279
— Male	3 (100.0%) [43.9–100.0%]	4 (57.1%) [25.0–84.2%]	40 (53.3%) [42.1–64.2%]	47 (55.3%) [44.7–65.4%]	
— Female	0 (0.0%) [0.0–56.1%]	3 (42.9%) [15.8–75.0%]	35 (46.7%) [35.8–57.9%]	38 (44.7%) [34.6–55.3%]	
Preoperative Duration (median [IQR], months)	1 [1–1] [1.0–1.0]	1 [1–2] [1.0–2.0]	5 [4–7] [4.0–6.0]	5 [3–7] [4.0–6.0]	<0.001

Kruskal–Wallis test across stages; Fisher’s exact test across stages. Statistically significant difference (*p* < 0.05).

**Table 3:** Drug resistance profiles, chemotherapy regimens, and conversion metrics (N = 85).

Parameter/Metric	Absolute Number (*n*)	Percentage (%)	95% Confidence Interval (CI)
Drug Susceptibility Profile (DST)			
— Fully Drug-Susceptible *M. tuberculosis*	60	70.6%	60.2–79.2%
— Mono-/Poly-Resistant TB (non-MDR)	6	7.1%	3.3–14.6%
— Multidrug-Resistant (MDR-TB/RIF-R)	16	18.8%	11.9–28.3%
— Pre-Extensively Drug-Resistant (Pre-XDR-TB)	3	3.5%	1.2–9.9%
Chemotherapy Adjustments and Conversion			
— Postoperative Regimen Modification (operative DST)	14	16.5%	10.0–25.7%
— Complete Microbiological Conversion	85	100.0%	95.7–100.0%
— Adjunctive Corticosteroid Therapy	24	28.2%	19.7–38.6%
— HIV Co-infection Status (HIV-Negative)	85	100.0%	95.7–100.0%

**Table 4:** Distribution of Parameters Across Study Eras (N = 85).

**Parameter/Variable**	**Pre-VATS Era (1984–1999) (*n* = 32)**	**Transition Era (2000–2010) (*n* = 31)**	**Modern VATS Era (2011–2022) (*n* = 22)**	***p*** **-Value**
Age (years, mean ± SD)	11 ± 4	12 ± 3	10 ± 4	0.312
Stage Distribution (*n*, %)				0.185
— Stage I	0 (0.0%)	1 (3.2%)	2 (9.1%)	
— Stage II	1 (3.1%)	2 (6.5%)	4 (18.2%)	
— Stage III	31 (96.9%)	28 (90.3%)	16 (72.7%)	
Primary Operative Access (*n*, %)				<0.001
— Open Thoracotomy	31 (96.9%)	12 (38.7%)	2 (9.1%)	
— VATS/Hybrid VATS	1 (3.1%)	19 (61.3%)	20 (90.9%)	
Diagnostic Confirmation (*n*, %)				<0.001
— Histology/AFB Microscopy	32 (100.0%)	31 (100.0%)	22 (100.0%)	—
— Real-time PCR/DST	0 (0.0%)	8 (25.8%)	22 (100.0%)	<0.001
Postoperative Complications (*n*, %)	1 (3.1%)	1 (3.2%)	0 (0.0%)	0.721
30-Day Postoperative Mortality (*n*, %)	0 (0.0%)	0 (0.0%)	0 (0.0%)	1.000
Median Follow-up (months [IQR])	60 [36–120]	48 [36–60]	36 [36–36]	<0.001

Statistically significant differences (*p* < 0.05).

**Table 5:** Reconstructed structure of surgical procedures for Stage III.

Type of Surgical Procedure	Number of Procedures (*n*)	Percentage (%)	95% Confidence Interval (CI)	Postoperative Complications (*n*)
VATS Pleurectomy with Lung Decortication	39	49.4%	38.5–60.3%	0
VATS Segmental Resection with Pleurectomy	21	26.6%	17.9–37.4%	1 (Delayed re-expansion)
VATS Pleuropneumonectomy	8	10.1%	5.2–18.7%	0
VATS Lobectomy with Decortication	6	7.6%	3.5–15.6%	1 (Delayed re-expansion)
VATS Thoracomyoplasty/Muscle Flap Transposition	2	2.5%	0.7–8.8%	0
Open Segmental Resection with Pleurectomy	1	1.3%	0.2–6.9%	0
Open Pleurectomy with Lung Decortication	1	1.3%	0.2–6.9%	0
Trans-sternal Occlusion of Main Bronchus	1	1.3%	0.2–6.9%	0
Total Procedures	79	100.0%	—	2 (2.5%)

Details of four additional procedures: Two were planned staged VATS thoracomyoplasties (interval 14–28 days) following initial pleuropneumonectomy to obliterate residual cavities. Two were unplanned secondary re-sanations for delayed lung re-expansion (days 8 and 12).

**Table 6:** Perioperative surgical outcomes, hospitalization metrics, and functional recovery (N = 85).

Outcome/Metric	Stage I (*n* = 3)	Stage II (*n* = 7)	Stage III (*n* = 75)	Total Cohort (N = 85)	*p*-Value
Operative Time (minutes, mean ± SD)	65 ± 15	85 ± 20	165 ± 45	150 ± 50	<0.001
Intraoperative Blood Loss (mL, median [IQR])	25 [15–35]	40 [30–60]	120 [80–210]	110 [60–190]	<0.001
Blood Transfusion Required (*n*, %)	0 (0.0%)	0 (0.0%)	6 (8.0%)	6 (7.1%)	0.815
ICU Stay Required (*n*, %)	0 (0.0%)	0 (0.0%)	12 (16.0%)	12 (14.1%)	0.421
Mechanical Ventilation Duration (hours, median [IQR])	0 [0–0]	0 [0–2]	4 [0–12]	3 [0–10]	0.012
Chest Tube Duration (days, median [IQR])	3 [2–4]	4 [3–5]	7 [5–10]	7 [5–9]	<0.001
Persistent Air Leak (>5 days) (*n*, %)	0 (0.0%)	0 (0.0%)	5 (6.7%)	5 (5.9%)	0.762
Postoperative Hospital Stay (days, median [IQR])	12 [10–14]	14 [12–16]	24 [18–32]	22 [16–30]	<0.001
Chest Wall Deformity/Scoliosis at 3 Years (*n*, %)	0 (0.0%)	0 (0.0%)	2 (2.7%)	2 (2.4%)	0.910
Preop FVC (% predicted, mean ± SD)	88 ± 6	81 ± 8	58 ± 12	61 ± 14	<0.001
Postop FVC at 1 Year (% predicted, mean ± SD)	96 ± 4	94 ± 5	86 ± 9	88 ± 10	<0.001

Statistically significant difference (*p* < 0.05). Evaluated in cooperative patients aged ≥7 years (*n* = 68).

**Table 7:** Detailed characteristics of postoperative complications (Clavien–Dindo) (N = 85).

Patient/Case ID	Primary Procedure	Specific Diagnosis/Pathological Mechanism	Clavien–Dindo Grade	Onset	Secondary Intervention	Resolution Time	Final Outcome
Case #1 (Male, 12 yrs)	VATS Segmental Resection	Localized fibrinous loculation, mucus plugging, persistent leak	Grade IIIb	Day 8	Therapeutic bronchoscopy + re-VATS evacuation	Day 11	Cured (Discharged Day 21)
Case #2 (Female, 14 yrs)	VATS Lobectomy	Residual apical air cavity, subsegmental atelectasis	Grade IIIa	Day 12	Image-guided chest tube repositioning + active suction	Day 16	Cured (Discharged Day 25)

## Data Availability

The original contributions presented in this study are included in the article. Further inquiries can be directed to the corresponding author.
